# Greenhouse Gas Emission Efficiencies of World Countries

**DOI:** 10.3390/ijerph17238771

**Published:** 2020-11-25

**Authors:** Levent Kutlu

**Affiliations:** Department of Economics and Finance, University of Texas Rio Grande Valley, Edinburg, TX 78539, USA; levent.kutlu@utrgv.edu

**Keywords:** eco-efficiency, greenhouse gas, Kyoto protocol, stochastic frontier analysis, pollution

## Abstract

Greenhouse gas emissions have increased rapidly since the industrial revolution. This has led to an unnatural increase in the global surface temperature, and to other changes in our environment. Acknowledging this observation, the United Nations Framework Convention on Climate Change started an international environmental treaty. This treaty was extended by Kyoto protocol, which was adopted on 11 December 1997. Using the stochastic frontier analysis, we analyze the efficiencies of countries in terms of achieving the lowest greenhouse gas emission levels per GDP output in the years between 1990–2015. We find that the average greenhouse gas emission efficiencies of world countries for the time periods 1990–1997, 1998–2007, 2008–2012, and 2013–2015 are 82.40%, 90.37%, 89.54%, and 84.81%, respectively. Moreover, compared to the 1990–1997 period, 92.50%, 79.51%, and 59.84% of the countries improved their greenhouse gas emission efficiencies in the 1998–2007, 2008–2012, and 2013–2015 periods, respectively. Hence, the Kyoto protocol helped in increasing greenhouse emission efficiency. However, this efficiency-boosting effect faded away over time.

## 1. Introduction

Greenhouse gases (GHG) have important environmental and health consequences. For example, they impede upon the process of infrared radiation leaving the earth’s atmosphere, which warms the surface of the planet. This can disturb the delicate balance in nature. In particular, warmer weather would worsen a variety of disasters and lead to disturbances including: reduced access to clean drinking water, more acidic oceans, droughts, floods, heat waves, storms, dust storms, etc. The increase in the intensity of rains may lead to the contamination of drinking water and increase in mold infestation, which in turn may lead to a variety of relate diseases. Moreover, greenhouse gases may directly contribute to respiratory diseases due to air pollution. 

Related to these concerns, in 1992, the United Nations Framework Convention on Climate Change (UNFCCC) opened for signatures for an international environmental treaty, which entered in force in 1994. The UNFCCC aimed at stabilizing greenhouse gas emissions to avoid their dangerous effects. Although the treaty does not have any enforcement mechanism, it describes how international protocols may be negotiated between relevant parties. On 11 December 1997, the Kyoto protocol established legally binding responsibilities for developed countries regarding their greenhouse gas emissions. The protocol consisted of two commitment periods, 2008–2012 and 2013–2020. Late in 2015, the Paris agreement was adopted, which effectively replaced the Kyoto protocol and aimed to control global greenhouse emissions toward the objective of lowering global warming to below 2 °C above the pre-industrial levels of 1750, and pursued efforts to limit it to 1.5 °C.

Despite these global attempts to slowdown greenhouse gas emissions, the amount of greenhouse gas emissions increased about 41.1% between 1990–2016. In line with this, the average global surface temperature increased about 0.57 °C in this time period. To put some of the consequences of global climate change in numbers, we note that relative to 1981–2010 averages, the Arctic sea ice is declining at a rate of 12.85% per decade; and based on 1993–2020 satellite data, the sea levels are increasing 3.3 mm per year. We obtained the climate data from https://climate.nasa.gov/vital-signs and https://www.climatewatchdata.org.

The GHG emission levels are closely related to the production of countries, which may be measured by their (real) gross domestic products (GDP). Hence, it is essential to understand the production technologies of countries that represent the production process of good (e.g., GDP) and bad (e.g., pollutants) outputs. There is a reasonably large literature on modeling production technologies and performance benchmarking in the presence of undesirable (bad) outputs. Two paradigms are prevalent in the literature, one involving parametric models that would be estimated using econometrics (e.g., stochastic frontier models); and one using mathematical programming methods (e.g., data envelopment analysis (DEA)). We utilize the parametric approach, i.e., stochastic frontier analysis. 

The literature has myriads of papers that use variations of DEA approach. Some examples to such studies that are related GHG emissions include [1,2,3,4,5,6]. Mukherjee [1] applied a directional distance function approach to measure energy efficiency of the manufacturing sector in India; and concludes that the states may increase output while reducing energy inputs through technical efficiency improvements. Molinos-Senante et al. [2] use DEA to measure the potential of reducing GHG emission in Spanish wastewater treatment plants. Using a DEA approach, Sueyoshi and Wang [3] examine the operational and environmental efficiencies of petroleum firms in the US. In their analysis, they assume that GHG produced by these companies is an undesirable output. They find that the integrated companies outperformed the independent companies. Vlontzos et al. [4] use DEA and directional distance function approaches to determine efficiency with both GHG emissions and without such emissions. Emrouznejad et al. [5] suggest an inverse DEA model for optimally allocating CO_2_ emissions quota in the Chinese manufacturing industries. Using an inverse DEA approach, Wegener and Amin [6] examine GHG emission efficiency in the oil and gas sector. They argue that there is a room for efficiency improvement. Among others, see also [7,8,9,10,11,12,13,14,15,16,17,18,19,20,21,22,23,24] for studies that model production technology with desirable (good) and undesirable (bad) outputs. Moreover, Dakpo et al. [25] provide an excellent literature review on modeling pollution-generating technologies.

Examples to the studies that estimate GHG emission efficiency (with different definitions) for the world countries include [15,16,18,21]. In line with these studies, we estimate the greenhouse gas emission efficiencies of 122 countries between 1990–2015. The GHG emission efficiency is a measure of how successful the countries are in terms of keeping their GHG emission levels low relative to their production levels. In particular, we assume that the countries try to minimize GHG emission to real gross domestic product (GDP) ratio given their inputs; and using stochastic frontier analysis techniques, we estimate the radial distance of observed value and frontier (minimum) value of this ratio.

We find that the GHG emission efficiencies of countries mostly increased after the Kyoto protocol adopted. However, this positive effect on efficiency decayed especially in the second commitment period. The average and median GHG emission efficiencies are 87.22% and 87.81%, respectively. The pre-Kyoto (1990–1997), pre-first commitment (1998–2007), first commitment (2008–2012), and second commitment (2013–2015) average GHG emission efficiencies are 82.40%, 90.37%, 89.54%, and 84.81%, respectively (although the second commitment period is 2013–2020, our data ends in 2015). The reduction of efficiency in the second commitment period suggests that countries may not be as enthusiastic as in earlier days of the protocol.

## 2. Changes in Temperature, CO_2_ Equivalent Emission, Population, and Real GDP

**Figure 1:**
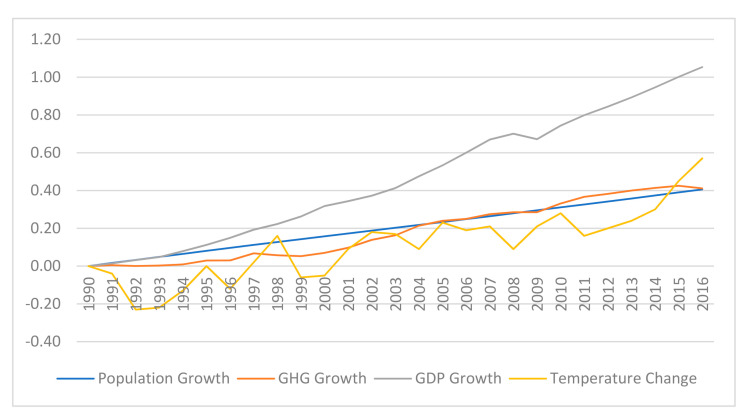
Historical World Data for Population, GHG Emission, Real GDP, and Temperature.

Figure 1 shows the changes in the global land-ocean temperature index (NASA’s Goddard Institute for Space Studies) relative to 1990 and growths of the total global GHG emission, global population, and global real (2010 dollars) GDP relative to 1990. Between 1990–2016, the global surface temperature increased 0.57 °C (between 1970–2016 the increase is 1 °C); the global GHG emission increased 41.1%; the global population increased 40.6%; and the global real GDP increased 105.4%. Therefore, the global GHG emission increased at similar rates with the global population and the global real GDP increased at a much faster rate. Based on this, we can either measure the GHG emission efficiencies of countries for given output (real GDP) levels or population. Since GDP is related to production, we prefer measuring efficiency relative to real GDP.

## 3. Materials and Methods

The GHG emission efficiency measure assumes that a country minimizes GHG emission to output (real GDP) given input levels. Then, the GHG emission efficiency is measured by the radial distance of observed GHG/GDP to the GHG/GDP possibility frontier, which represents the lowest amount of GHG/GDP possible for given input levels. The country specific GHG emission efficiency estimates are based on a benchmarking method—the so-called stochastic frontier analysis—which assumes that the inefficiency is an unobserved random variable that represents the radial distance from the relevant frontier. In this section, we first describe our dataset; then we describe our estimation methodology; and finally, we present the estimation results. 

### 3.1. Data

The GDP data is obtained from the International Monetary Fund (IMF) website (https://www.imf.org/), which is measured in billions of constant 2011 international dollars. The pollution output is the total greenhouse gas emissions including land use change and forestry (LUCF) measured in tonnes of CO₂ equivalents based on 100-year global warming potential factors for non-CO₂ gases. This dataset is obtained from climatewatchdata.org. (http://cait.wri.org and https://www.climatewatchdata.org) We dropped countries with average GHG emission levels that are smaller than 10,000 tonnes, as these countries are probably very different from the rest of the countries in terms of their production structures and scales. The input variables are labor (L), capital (K), and energy (E). The labor input is the total labor force, which is obtained from the World bank website; the capital input (sum of government, private, and public-private capital stocks) is measured in billions of constant 2011 dollars, which is obtained from the IMF website; and the energy input is the energy use in tonnes of oil equivalent, which is obtained from the World bank website. The population (POP) data is obtained from the World Bank website. (https://data.worldbank.org) The final dataset includes 122 countries and covers the years between 1990–2015. In Table 1, we provide descriptive statistics of our data set.

### 3.2. Econometric Model

In the efficiency analysis literature, there are two widely used methods: data envelopment analysis and stochastic frontier analysis. In this study, we use the latter. Stochastic frontier models were introduced by Aigner et al. [26] and Meeusen andvan den Broeck [27]. Jondrow et al. [28] presented a way to estimate firm specific technical efficiency. However, these models do not assume time-varying efficiency. Among others, [29,30,31,32,33,34,35] exemplify stochastic frontier models that allow time-varying efficiency (see Kumbhakar and Lovell [36] for an excellent literature review on earlier stochastic frontier models). In the panel data context, Greene [37,38] criticizes these models for not disentangling heterogeneity and efficiency, and argues that if the productive unit heterogeneity is not controlled in the estimation, it can be confused with inefficiency. He suggests controlling heterogeneity by panel unit specific fixed effects or random effects; and presents computationally feasible ways to obtain parameter estimates. He calls these models true fixed effects (TFE) and true random effects (TRE), respectively (see also [39,40,41] for studies that disentangle heterogeneity and efficiency; see [42] for more details about heterogeneity in stochastic frontier models). In this study, we use the true fixed effects model of Greene. McCarthy and Kutlu [43] illustrate negative consequences of ignoring heterogeneity via an empirical example in the airport efficiency context. Since in our setting GHG to real GDP ratio is minimized, our model is akin to a stochastic cost frontier model. Hence, below, we summarize the true fixed effects model in the context of GHG emission efficiency, which is similar to a stochastic cost frontier model.

Consider the following stochastic frontier function:

(1) $$y_{it}=\alpha_{i}+x_{it}^{\prime }\beta +v_{it}+u_{it},$$

where $y_{it}=\ln(GHG_{it}/GDP_{it})$ is the logarithm of ratio of GHG and GDP for country i at time t; $\alpha_{i}$ is the country fixed effects; $x_{it}$ is a vector of exogenous frontier variables; $v_{it}\sim N(0,\sigma_{v}^{2})$ is the usual error term; and $u_{it}=\exp(z_{it}^{\prime }\gamma )u_{it}^{*}$ so that $u_{it}^{*}\sim N^{+}(0,1)$ and $z_{it}$ is a vector of exogenous variables that explain GHG emission efficiency. Here, N(.,.) and $N^{+}(.,.)$ are normal and half-normal distributions, respectively. In line with Greene [19,20], we assume that $v_{it}$ and $u_{it}^{*}$ are independently and identically distributed. We estimate the efficiency by:

(2) $${\mathrm{Eff}}_{\mathrm{it}}=E[\exp(-u_{it})|\varepsilon_{it}],$$

where $\varepsilon_{it}=v_{it}+u_{it}$ is the composed error term. An alternative and widely used estimator of efficiency is given by:

(3) $${\mathrm{Eff}}_{\mathrm{it}}=\exp(-E[u_{it}|\varepsilon_{it}])$$

In practice, when estimating efficiency $\varepsilon_{it}$ is replaced by ${\hat{\varepsilon }}_{it}=y_{it}-{\hat{\alpha }}_{i}-x_{it}^{\prime }\hat{\beta }$ where ${\hat{\alpha }}_{i}$ and $\hat{\beta }$ are the corresponding parameter estimates.

### 3.3. Empirical Model

In line with a conventional production function, we assume that the GHG to GDP ratio is a function of inputs: labor, capital, and energy. Moreover, we include (logarithm of) population as a control variable. Among others, the population variable would control for non-labor force factors that can contribute to the production. Additionally, population controls for size of a country. As described in the econometric model section, we also control for the country fixed effects in order to control heterogeneity so that our efficiency measure does not confuse country specific fixed factors (technological or other time-invariant differences) with inefficiency.

When modeling the distribution of inefficiency, we considered three different models. In the first model, the distribution of the inefficiency term is modeled via the trend term. In the second model, the distribution is modeled via two dummy variables each corresponding to an important time period: D1998_2007 and D2008_2015. The first dummy variable represents the time period between adoption date (11 December 1997) and the first commitment period (1998–2007) (since 11 December 1997 is close to 1998, we start the first commitment period from 1998); and the second (2008–2015) dummy variable represents the first and second commitment periods (note that our dataset ends in 2015). In the third model, D2008_2012 and D2013_2015 dummy variables represent the first and second commitment periods, respectively.

## 4. Results

The estimation results are given in Table 2. For all three models, most of the frontier parameters are statistically significant at 0.01 or 0.001 levels. Frontier parameter estimates are similar. In particular, the pairwise correlations of frontier parameters are above 0.99. The signs of inefficiency parameters are not conflicting with each other. For all models, we find that the GHG emission efficiency increased after Kyoto protocol. Model 2 and Model 3 estimates suggest that although there is efficiency gain after Kyoto protocol, in the second commitment period the efficiency gain is smaller relative to earlier years of Kyoto protocol. Hence, it seems that initial efficiency gain effect of Kyoto protocol has faded away over time. In what follows, we will use Model 3 as our benchmark model. However, this positive effect on efficiency decayed especially in the second commitment period. The average and median GHG emission efficiencies are 87.22% and 87.81%, respectively. Moreover, the average GHG emission efficiencies for the time periods 1990–1997, 1998–2007, 2008–2012, and 2013–2015 are 82.40, 90.37%, 89.54%, and 84.81%, respectively. In line with this, compared to the 1990–1997 period, 92.50%, 79.51%, and 59.84% of the countries improved their GHG emission efficiency levels in the 1998–2007, 2008–2012, and 2013–2015 periods, respectively. Finally, compared to the 1990–1997 period, 61.67%, 59.07%, and 45.90% of the countries improved their GHG emission efficiency levels at least 5 percentage points in the 1998–2007, 2008–2012, and 2013–2015 periods, respectively.

**Figure 2:**
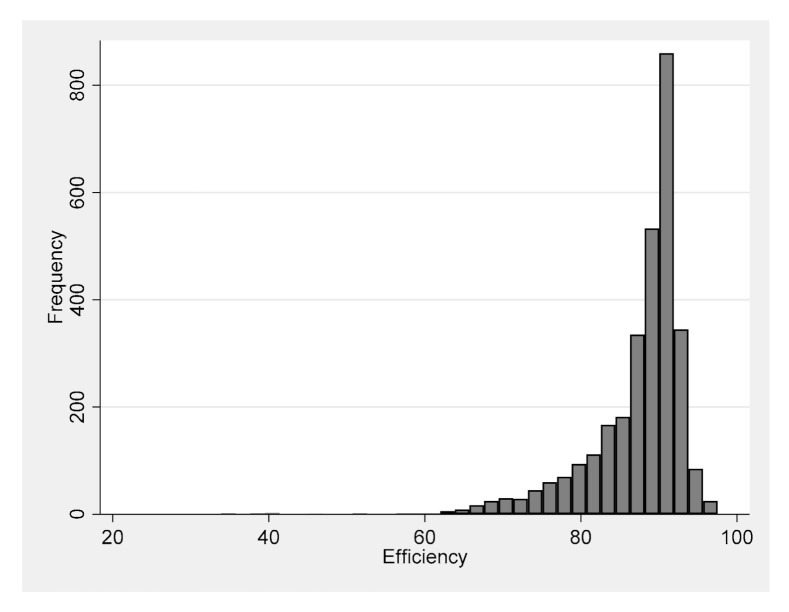
Histogram of GHG Emission Efficiency Estimates.

While the mean and median efficiency information are useful, to have a better idea about the distribution of the GHG emission efficiencies, we provide the histogram of GHG emission efficiency estimates in Figure 2. As it can be seen from the histogram, for a large number of observations the efficiency is at least 80%. In particular, 43.80% of the observations would have at least 90% efficiency; 88.02% of the observations would have at least 80% efficiency; and 97.24% of the observations would have at least 70% efficiency.

In order to give a better idea about country-specific nature of GHG emission efficiencies, in Table 3, we present the average GHG emission efficiency levels of countries for different time periods. In the table, we show the European Union countries in bold font. At the beginning of 2005, the European Union member countries launched a CO_2_ emissions trading scheme [5]. Out of 22 European Union countries in our sample, 7 of the countries (Austria, Croatia, Finland, Greece, Italy, Portugal, and Spain) had lower (average) GHG emission efficiency levels in 2005–2015 period compared to 1990–2004 period. Croatia showed the most dramatic efficiency change (from 90.74–83.25%). For other countries, the decrease in efficiency ranged from 0.67–2.61 percentage points. Compared to the pre-Kyoto period (1990–1997), in the pre-first commitment period (1998–2007), all European Union countries in our sample improved their average GHG emission efficiencies. In the first commitment period (2008–2012), only Croatia performed worse. This is partially due to the fact that Croatia was already somewhat efficient to begin with. For the full sample time period, on average, Spain is the most GHG emission efficient European Union country.

## 5. Discussion

In this section, we compare our model and findings with some of the related studies’ models and findings.

Herrala and Goel [15] examine global CO_2_ efficiency by employing a stochastic frontier analysis of 170 countries in 1997 and 2007. In contrast to our model, the dependent variable of their stochastic frontier models is the logarithm of CO_2_ emission. Note that we consider total CO2 equivalent GHG emissions rather than CO_2_. Hence, their model ignores other greenhouse gas pollutants. The frontiers of their models include the logarithm of GDP and some control variables such as logarithms population and area. However, unlike our model, their model does not include input variables for production. Hence, their measure of efficiency ignores input variables. Their findings suggest that relative to 1997 the average CO_2_ emission efficiencies of countries increased in 2007. This is in line with our findings. However, their average efficiency estimates are much lower than ours. In particular, the average of their efficiency estimates range between 40–64% depending on year and the variation of their model. These average efficiency values seem to be somewhat low compared to conventional efficiency studies. This may be due to the fact that the authors do not control for heterogeneity of countries as suggested by Greene [19,20]. Greene argues that when heterogeneity is ignored it may be confused with inefficiency.

Robaina-Alves et al. [18] examine GHG emission efficiency of 26 countries between 2000–2011. The dependent variable in their model is the ratio of real GDP and GHG emissions, which is similar to ours. Hence, in their scenario they assume that the countries try to maximize this ratio. They estimated their model using a variety of different estimation methods (i.e., maximum likelihood estimation method, generalized maximum entropy, generalized cross-entropy). In contrast to our model, their model has four input variables and assumes the Cobb-Douglas functional form, which is not commonly used in the stochastic frontier literature due to the fact that this is not a flexible functional form. In line with our paper, this paper also examines the effects of the Kyoto protocol by comparing 2000–2004 and 2005–2011 periods. They argue that Hungary, Slovenia, Portugal, and Ireland had significant improvements in terms of their efficiency rankings compared to first time period (i.e., 2000–2004). Additionally, within their sample countries, they state that Sweden, Latvia, UK, Portugal, and Cyprus are the six most efficient countries.

Valadkhani et al. [21] examine environmental and economic efficiencies of world’s major polluters for 2002, 2007, and 2011. In contrast to us, they use a multiplicative environmental data envelopment analysis approach to measure efficiency, which is a non-parametric method. They find that for most of the countries the overall efficiency scores increased between 2002–2011. However, they do not provide a clear answer in terms of overall environmental efficiency. In contrast to our study, which considers 122 countries over 26-year period, Valadkhani et al. [21] consider 46 countries for a three-year period. Their method and concentration are different; and so, our study complements theirs.

Another related study is Jin and Kim [16], which study the carbon emission efficiency of 21 emerging countries between 1995–2016. Similar to us, they estimate a true fixed effects stochastic frontier model where the dependent variable is carbon emission amount and frontier variables are energy consumption, capital, labor force, and economic complexity index. Hence, their efficiency measure purely concentrates on carbon emission rather than ratio of GHG to real GDP. Unlike us, they use the Cobb-Douglas functional form for estimations, which is not a flexible functional form. Since the frontier of their production function is missing the second order terms from a Translog functional form, if these second order terms are relevant, dropping them may have contaminated the inefficiency estimates. Their efficiency estimates for 21 countries range between 70.9–91.8%, which seems to be a reasonable.

## 6. Conclusions

We examined the GHG emission efficiencies of world countries between 1990–2015. This is a particularly interesting time period because of increased global awareness about consequences of GHG emissions. Adoption of the Kyoto protocol is one of the evidences of such awareness. We showed that the adoption and implementation of the Kyoto protocol helped the environment in terms of keeping their GHG emission relative to their (real) GDP. However, over time the extend of efficiency improvement decreased. This indicates that world countries must find ways to keep their enthusiasm for greenhouse emission reduction at high levels.

Finally, note that in this paper we concentrated on GHG emission efficiency rather than the total GHG emission amounts. Hence, for given input levels, as long as the GHG emission grows slower than real GDP, the efficiency would improve. Therefore, although, in general, the GHG emission levels increased over time, we still observed some increase in efficiency levels. One potential way to improve the GHG emission efficiency levels is investing in environment friendly production technologies, which can shift the GHG emission to the real GDP frontier. That is, this way they can produce the same amount of good output while lowering the bad output amount. The governments may set environmental taxes. This would introduce incentives for companies to research and invest in environmentally friendly technologies. Collected environmental taxes may be redistributed as subsidies in the form of grants, loans with low interest, procurement mandates, better tax treatments, etc. Caruso et al. [44] argue that individual well-being and public awareness may stimulate a greater demand for energy obtained from renewable sources [45], which in turn may improve GHG emission efficiency. Therefore, policymakers should not only use incentive/disincentive tools (e.g., subsidy and tax) but also promote public awareness about renewable-energy economies.

## Figures and Tables

**Table 1:** Descriptive statistics.

Variable	Unit	Mean	Std. Dev.	5th Perc.	Median	95th Perc.
GHG	Million tonnes	315.86	903.79	7.31	69.02	1266.66
GDP	Billion dollars	578.10	1611.86	11.08	110.66	2397.32
L	Million	22.09	77.10	0.49	4.79	67.37
K	Billion dollars	1264.48	3544.88	19.69	195.31	5692.87
E	Million kg oil equivalent	85.42	271.69	1.69	15.89	305.71
POP	Million	48.70	154.07	1.11	10.55	148.52
# of Obs.		3048				

**Table 2:** Estimation results for greenhouse gases (GHG) emission efficiency model.

	Model 1			Model 2			Model 3		
**ln(GHG/GDP)**	**Coeff.**	**S.E.**		**Coeff.**	**S.E.**		**Coeff.**	**S.E.**	
ln(L)	0.0315	0.1039		0.0353	0.1050		0.0293	0.1063	
ln(K)	−0.1436	0.0302	***	–0.1439	0.0301	***	–0.1440	0.0302	***
ln(E)	0.3628	0.0324	***	0.3642	0.0325	***	0.3634	0.0325	***
T	−0.0191	0.0014	***	–0.0259	0.0013	***	–0.0264	0.0016	***
0.5 × ln(L)^2^	−0.1485	0.0404	***	–0.1547	0.0407	***	–0.1549	0.0408	***
0.5 × ln(K)^2^	0.0893	0.0278	***	0.0967	0.0274	***	0.0970	0.0274	***
0.5 × ln(E)^2^	−0.1413	0.0452	**	–0.1422	0.0451	**	–0.1424	0.0453	***
0.5 × T^2^	−0.0019	0.0002	***	–0.0022	0.0002	***	–0.0023	0.0003	***
ln(L) × ln(K)	0.0133	0.0187		0.0143	0.0186		0.0146	0.0187	
ln(L) × ln(E)	0.1898	0.0302	***	0.2006	0.0305	***	0.2014	0.0307	***
ln(L) × T	−0.0061	0.0011	***	–0.0060	0.0011	***	–0.0060	0.0011	***
ln(K) × ln(E)	−0.0423	0.0312		–0.0535	0.0308		–0.0542	0.0308	
ln(K) × T	0.0045	0.0010	***	0.0041	0.0010	***	0.0041	0.0010	***
ln(E) × T	−0.0007	0.0012		–0.0002	0.0012		–0.0002	0.0012	
ln(POP)	−0.0377	0.1204		–0.0319	0.1214		–0.0227	0.1234	
σ_v_									
Constant	−3.4049	0.0412	***	–3.4925	0.0662	***	–3.5034	0.0803	***
σ_u_									
T	−0.2251	0.0277	***	-	-	-	-	-	-
D1998_2007	−	-	-	-	-	-	–1.3956	0.1948	***
D2008_2012	−	-	-	-	-	-	–1.2147	0.3915	**
D1998_2012	−	-	-	–1.3935	0.1931	***	-	-	-
D2013_2015	−	-	-	–0.5455	0.2335	*	–0.4154	0.3355	
Constant	−4.5389	0.2840	***	–2.6561	0.1588	***	–2.6648	0.1606	***
Ave. Efficiency	90.19			87.41			87.23		
Log-likelihood	495.9877			489.8614			490.0955		

Note: standard errors are given in parenthesis. * *p* < 0.05, ** *p* < 0.01, *** *p* < 0.001.

**Table 3:** Average GHG emission efficiency estimates.

COUNTRY	1990–1997	1998–2007	2008–2012	2013–2015	1990–2015	COUNTRY	1990–1997	1998–2007	2008–2012	2013–2015	1990–2015
Albania	80.42	90.84	91.48	87.26	87.34	Lithuania	74.60	91.02	91.89	90.00	85.86
Algeria	88.24	90.99	88.05	79.67	88.62	Luxembourg	74.57	91.57	90.28	89.54	85.86
Angola	73.29	88.76	93.79	93.16	85.17	Malaysia	68.62	86.09	94.00	95.65	82.85
Argentina	86.81	90.12	89.54	86.13	88.62	Malta	84.81	91.53	88.49	83.99	88.17
Armenia	66.70	91.76	91.86	87.49	83.42	Mauritius	90.87	90.04	86.85	78.33	88.73
Australia	84.05	90.17	90.68	89.78	88.34	Mexico	88.11	91.32	87.47	81.38	88.44
**Austria**	86.69	90.86	89.26	84.18	88.50	Moldova	81.73	90.16	91.29	89.75	87.65
Azerbaijan	79.66	87.99	92.41	88.22	86.23	Mongolia	77.37	89.08	92.78	93.57	86.43
Bahrain	88.21	90.83	88.48	80.12	88.67	Morocco	90.02	90.79	85.86	77.79	88.52
Bangladesh	80.54	90.81	91.45	88.99	87.50	Mozambique	77.78	91.17	91.64	86.23	86.58
Belarus	71.78	91.11	92.22	82.26	84.44	Myanmar	72.96	90.40	92.47	91.18	85.29
**Belgium**	81.77	90.86	91.17	87.74	87.76	Namibia	82.73	91.18	89.00	89.21	88.10
Benin	78.75	91.12	91.14	88.37	86.95	Nepal	77.73	89.64	92.86	89.46	86.46
Bolivia	83.06	90.90	89.24	89.50	87.95	**Netherlands**	80.06	91.58	90.76	85.48	87.18
Botswana	73.28	85.12	94.75	91.03	83.73	New Zealand	86.93	90.85	89.22	83.76	88.51
Brazil	80.33	88.85	93.07	91.92	87.21	Nicaragua	77.38	88.39	89.86	95.77	85.75
**Bulgaria**	85.20	91.29	88.96	84.47	88.33	Niger	-	91.82	88.08	84.41	89.59
Cambodia	83.08	89.82	91.49	90.42	89.29	Nigeria	69.50	90.17	93.22	90.93	84.23
Cameroon	87.59	90.81	87.37	81.83	88.38	North Macedonia	88.32	90.09	89.32	84.64	88.93
Canada	87.43	90.35	89.93	83.97	88.63	Norway	76.27	90.97	91.53	88.51	86.27
China	80.99	90.80	91.16	89.60	87.63	Oman	92.26	90.15	81.54	65.56	87.13
Colombia	84.07	88.41	90.91	94.01	87.97	Pakistan	88.37	91.17	87.46	78.71	88.53
Congo, Dem. Rep.	89.76	87.94	89.48	90.08	89.00	Panama	80.37	90.65	91.56	89.68	87.46
Congo, Rep.	80.92	89.72	89.61	89.49	86.87	Paraguay	89.70	91.02	84.08	76.27	88.03
Costa Rica	38.17	85.65	94.28	95.74	72.99	Peru	86.35	90.50	89.38	87.88	88.74
Cote d’Ivoire	82.56	89.33	87.28	86.59	86.53	Philippines	88.06	88.86	88.40	92.09	88.77
**Croatia**	89.85	91.19	84.33	70.55	87.74	**Poland**	75.08	90.97	92.31	86.79	85.86
**Cyprus**	84.78	91.20	89.92	82.82	88.22	**Portugal**	88.29	90.71	89.19	80.23	88.47
**Czech Republic**	82.38	90.90	90.78	87.96	87.92	Qatar	83.60	90.05	91.57	89.14	88.22
**Denmark**	80.19	90.99	90.87	89.44	87.46	Russian Federation	84.58	90.19	90.89	86.75	88.26
Dominican Republic	91.46	89.50	86.13	78.45	88.57	Saudi Arabia	90.28	90.84	85.55	76.93	88.49
Ecuador	83.19	90.92	90.48	88.00	88.13	Senegal	85.66	90.72	90.31	84.27	88.50
Egypt, Arab Rep.	87.70	90.93	88.74	80.50	88.62	Singapore	77.05	90.19	92.55	90.89	86.51
El Salvador	88.27	91.29	86.88	79.05	88.46	**Slovak Republic**	73.19	90.87	92.25	90.34	85.63
**Estonia**	80.36	91.52	89.62	82.16	86.64	**Slovenia**	81.60	91.98	88.36	83.27	87.09
Ethiopia	83.51	89.96	91.31	89.84	88.15	South Africa	88.24	91.10	87.69	79.08	88.54
**Finland**	86.35	91.35	88.44	76.24	87.51	**Spain**	87.74	90.47	89.68	83.97	88.73
**France**	81.65	91.10	90.54	87.98	87.72	Sri Lanka	70.06	91.32	92.32	90.55	84.66
Georgia	73.17	91.74	90.73	81.95	84.81	Sudan	91.13	89.75	85.74	81.75	88.75
**Germany**	84.62	91.35	89.68	83.93	88.10	Suriname	-	90.20	90.23	84.16	89.40
Ghana	82.74	85.14	93.61	87.15	86.23	**Sweden**	68.31	90.94	82.60	76.70	80.73
**Greece**	86.73	91.42	88.85	79.33	88.09	Switzerland	84.76	90.91	90.24	86.05	88.33
Guatemala	84.50	90.26	90.40	90.13	88.44	Syrian Arab Republic	85.66	91.73	88.73	69.47	87.41
Haiti	91.80	89.75	84.94	73.43	88.14	Tajikistan	83.48	90.28	89.98	85.70	87.68
Honduras	77.65	90.85	91.81	88.06	86.60	Tanzania	74.48	90.12	92.68	92.76	85.84
**Hungary**	83.88	90.96	90.47	86.29	88.15	Thailand	86.33	90.95	89.36	83.11	88.53
Iceland	80.54	90.85	91.18	88.87	87.51	Togo	87.84	90.96	87.61	81.48	88.53
India	79.91	91.03	91.29	88.40	87.31	Trinidad and Tobago	78.53	92.07	89.77	83.48	86.59
Indonesia	82.66	90.40	90.53	86.97	87.67	Tunisia	85.57	90.99	89.88	83.68	88.45
Iran, Islamic Rep.	89.39	91.04	86.95	73.24	88.27	Turkey	86.67	91.01	89.14	84.15	88.53
Iraq	64.08	92.91	90.16	82.14	82.27	Ukraine	85.46	90.13	90.45	83.36	88.16
**Ireland**	71.29	91.30	91.93	89.15	85.02	United Arab Emirates	82.70	91.41	89.98	84.85	87.81
Israel	86.92	90.84	89.19	84.93	88.64	**United Kingdom**	80.09	91.20	90.82	88.39	87.38
**Italy**	88.12	90.94	88.42	82.22	88.58	United States	83.79	91.37	90.03	84.49	87.99
Japan	89.37	91.33	87.04	71.36	87.60	Uruguay	94.83	82.96	78.80	63.24	84.35
Jordan	77.58	91.71	91.21	83.44	86.43	Uzbekistan	85.92	89.38	91.29	91.11	88.70
Kazakhstan	76.82	91.61	90.67	87.81	86.38	Venezuela, RB	83.73	89.44	91.97	84.63	87.86
Korea, Rep.	84.68	91.42	89.53	83.75	88.10	Vietnam	94.28	86.79	79.41	69.37	87.03
Kuwait	81.23	91.41	88.07	79.28	87.23	Yemen, Rep.	89.17	91.42	84.41	69.58	88.30
Kyrgyz Republic	89.16	89.87	76.44	81.51	86.29	Zambia	74.39	89.78	93.54	93.34	85.58
Lebanon	89.42	89.80	89.09	80.77	88.82	Zimbabwe	91.75	87.99	85.68	87.70	88.75
